# One-Stage Ear Reconstruction After Avulsion Injury, Using the Amputated Cartilage and a Retroauricular Transposition Flap

**Published:** 2010-01-18

**Authors:** Theodora Manoli, Patrick Jaminet, Armin Kraus, Hans-Eberhard Schaller, Frank Werdin, Nektarios Sinis

**Affiliations:** Clinic of Hand, Plastic, Reconstructive Surgery and Burn Unit, Eberhard Karls University of Tuebingen, BG Trauma Center, Tuebingen, Germany

## Abstract

**Introduction:** Auricular reattachment or reconstruction after traumatic ear loss remains a challenge for the plastic reconstructive surgeon. Because of the diverse accident mechanisms, no standard algorithms exist and several modalities have been proposed in the literature. **Methods:** A case of an innovative ear reconstruction of a partially avulsed ear is presented. The amputated cartilage was reattached after being deepithelized from the anterior skin. A transauricular-retroauricular random pattern flap was then harvested and used for anterior skin coverage. **Results:** The described technique provided a nice final result without the need of any further operations. **Conclusion:** In general, a microsurgical replantation should be applied when the circumstances allow. In other cases, especially in partial upper-ear amputations with severe skin contusions, the described technique should be considered as a safe, single-step approach with good final results.

The evolution of microsurgery introduced new challenging reconstructive methods also in the field of ear replantations that offer optimum results in a single procedure.^1^ However, auricle revascularization techniques require special microsurgical skills, which can be expected only in certain specialized centers. A sufficient venous drainage is often not possible, so that an intensive application of leeches and systemic heparinization is mandatory for the survival of the replanted ear. These can cause continuous or even uncontrolled bleeding, and several blood transfusions may be necessary. Hospitalization of the patient will be then prolonged significantly.^2^,^3^

Several nonmicrosurgical ear reattachment techniques have been described,^4^ including direct reattachment,^5^ composite grafting,^6^ pocket methods,^7^ and the use of periauricular tissue flaps such as the temporal fascia flap^8^^-^10 or the Baudet method.^11^^-^13 However, these techniques show a higher rate of partial necrosis or late deformity when compared with microsurgical techniques. On the other hand, nonmicrosurgical techniques count as safe methods, with almost no failure rates (excluding the direct ear reattachment).

In the current report, we present an innovative nonmicrosurgical reconstruction technique of a partially avulsed ear, using the amputated cartilage and a retroauricular transposition flap as a single-stage procedure.

## CASE REPORT

### Initial presentation

**Figure 1:**
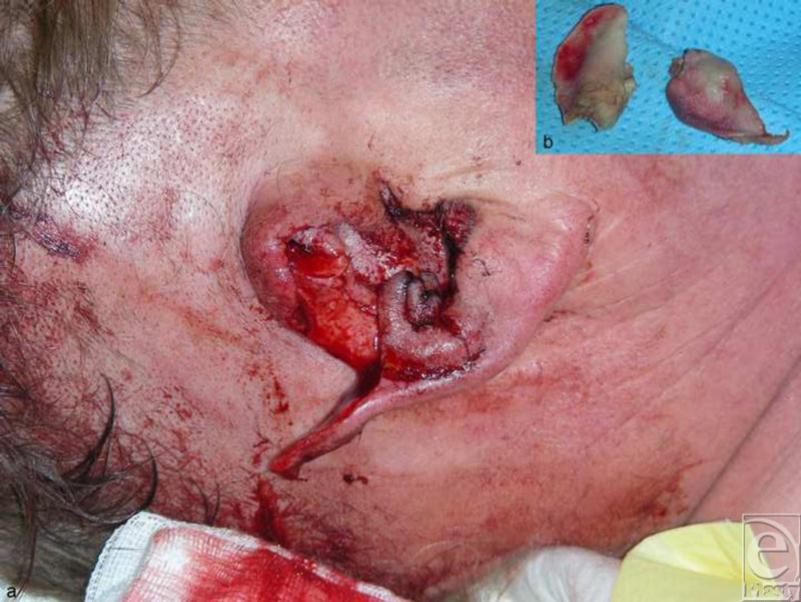
Initial presentation.

A 56-year-old male farmer presented in our department with a partial avulsion of the superior half of his right ear, after a fall from his tractor 3 hours earlier. The amputated auricle was transferred on ice. The wish of our patient was a possibly single-step, safe ear reconstruction, without significant reduction of size. The recovery period after operation should also be not much extended. The patient was transferred to the operation room approximately 4 hours after the accident. The severely contused amputated part consisted of the detached cartilage part, with its covering anterior skin between helix and concha, extending from the middle to the upper part of the pinna. The assessment of the remaining attached part of the ear revealed an intact posterior auricular skin, while the helix, the antihelix, and the concha were partially avulsed, connected to the auricle by just a thin caudal skin pedicle (Fig 1a).

## Operative method

**Figure 2:**
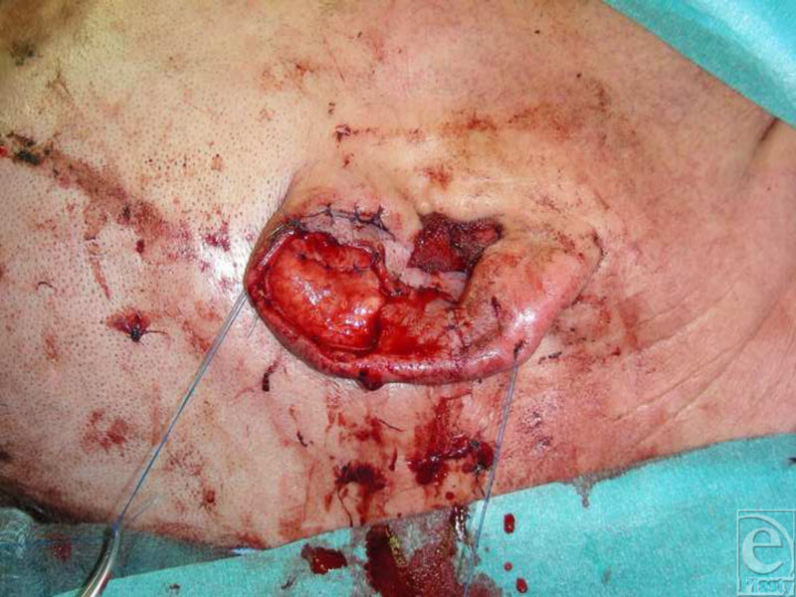
Reconstruction of avulsed parts and reattachment of the cartilage graft.

**Figure 3:**
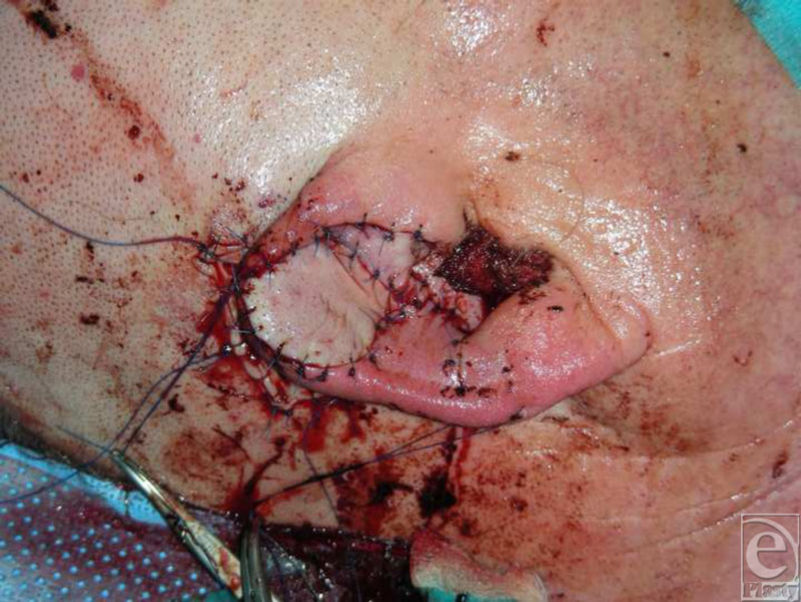
Postoperative result.

Because of severe contusions, the anterior skin was completely deepithelized from the amputated part (Fig 1b) in order to obtain a free cartilage graft. The avulsed parts of the helix and the concha were reconstructed by direct reattachment with 5-0 nylon sutures. The cartilage graft was then medially attached to the posterior skin and laterally to the reconstructed helix, with 5-0 polydioxanone sutures (Fig 2). Afterward, a trapezoid-shaped skin flap, approximately 1.5 × 2 cm, was elevated from the retroauricular region, remaining attached to the scalp medially at the retroauricular fold. The flap was then passed through a longitudinal transauricular incision between the replanted cartilage and the reconstructed concha. After adjusting its dimensions to the defect area, the flap was attached to the surrounding anterior skin with 5-0 nylon sutures. Taking into consideration the vascular anatomy of the ear, the incision was made between 2 constant vital ear arteries: the upper branch of the superficial temporal artery and the helical root perforator of the posterior auricular artery.^14^ Despite the long incision, we speculated that there would be no risk of postoperative ear ischemia due to the sparing of this vascular supply. The retroauricular defect was covered with a full-thickness skin graft from the right supraclavicular neck region, which was also attached with a 5-0 nylon continuous suture (Fig 3). The donor area at the right lateral neck was sutured intracutaneously with 3-0 nylon.

## Postoperative management

To improve the tissue blood circulation, infusion of hydroxyethyl starch 500 mL over 24 hours was supplied for 5 days. An intravenous antibiotic (cefazolin 2 g, 3 times a day) was applied for 7 days. The retroauricular compression bandage was removed on the fourth postoperative day. The initial swelling of the ear was treated by regular cooling measures. The patient was hospitalized for 10 days.

## Follow-up evaluation

**Figure 4:**
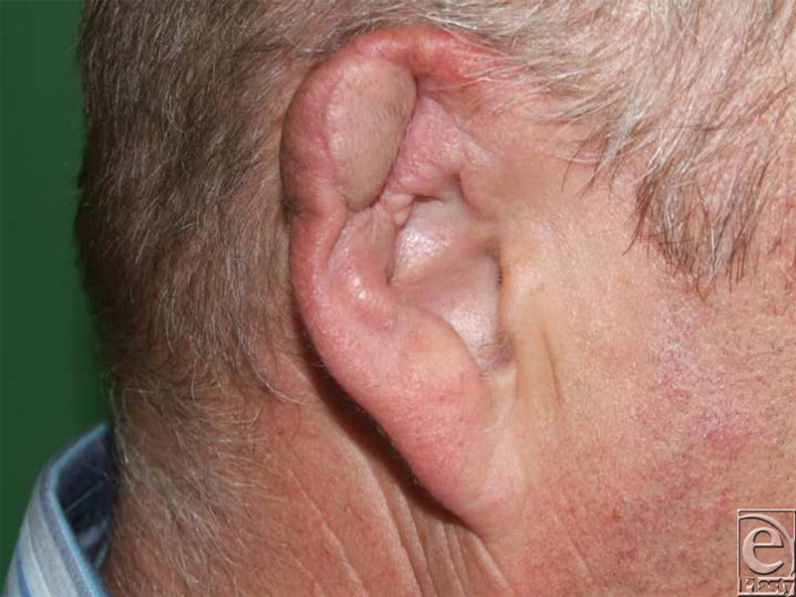
Nine weeks after operation.

Nine weeks after the operation, the ear appeared entirely healed. The flap region was slightly darker and thicker than the surrounding posterior skin. At the caudal, lateral flap corner, a slight contraction of the helix was observed. The retroauricular donor site was completely healed as well and was hardly distinguishable from the adjacent skin. Considering the accident mechanism, the patient was very satisfied with the final result (Fig 4).

## DISCUSSION

The reconstructive algorithm described above is based on the rich vascularization of the head region, which allows surgeons to make use of random pattern flaps to cover soft-tissue defects.^14^ The retroauricular flap used in this case could be considered as a reverse-Baudet technique.^12^ After removing the posterior skin of the amputated part, Baudet^12^ made big fenestrations in the cartilage to allow a sufficient connection of the anterior skin to the posterior area. A retroauricular flap, this time laterally pedicled, was elevated and the anterior skin of the amputated part was then sutured medially to the stump of the ear and laterally to the retroauricular flap. The Baudet technique gives satisfactory results^13^ in comparison with other nonmicrosurgical techniques, such as the pocket method^7^ and the temporal fascia flap,^8^^-^10 that lead to late shrinkage of the ear. However, a second operation is required to elevate the posterior ear, about 3 months later. Our technique is considered to be an one-stage operation, offering a fast convalescence with good final results. A drawback of our technique is that hair follicles may remain in the flap region. Our patient will have to shave this part, while a permanent hair removal can be achieved by photoepilation.^15^

## CONCLUSION

Microsurgical ear replantation^1^^-^3 still offers the best aesthetical results and should be applied when an arterial anastomosis is possible. Our proposed technique is a good alternative in cases where the posterior skin of the pinna stump is at least in its largest part maintained, when a microsurgical reattachment cannot take place, and when a safe, single-step procedure is preferred.
